# Effective access to health care in Mexico

**DOI:** 10.1186/1472-6963-14-S2-P42

**Published:** 2014-07-07

**Authors:** Juan Pablo Gutierrez, Sebastian Garcia-Saiso, German Fajardo-Dolci, Mauricio Hernandez-Avila

**Affiliations:** 1 Center for Evaluation Research & Surveys, National Institute of Public Health, Cuernavaca, Mexico; 2 Ministry of Health, Ciudad de Mexico, Mexico; 3 The Mexican Institute of Social Security, Ciudad de Mexico, Mexico; 4 Directorate-General, National Institute of Public Health, Cuernavaca, Mexico

## Background

Effective access measures are intended to reflect progress toward universal health coverage. This study proposes an operative approach to measuring effective access: in addition to the lack of financial protection, the willingness to make out-of-pocket payments for health care signifies a lack of effective access to pre-paid services.

## Materials and methods

Using data from a nationally representative health survey in Mexico, effective access at the individual level was determined by combining financial protection and effective utilization of pre-paid health services as required. The measure of effective access was estimated overall, by sex, by socioeconomic level, and by federal state for 2006 and 2012.

## Results

In 2012, 48.49% of the Mexican population had no effective access to health services. Though this represents an improvement since 2006, when 65.9% lacked effective access, it still constitutes a major challenge for the health system. Effective access in Mexico presents significant heterogeneity in terms of federal state and socioeconomic level.

## Conclusions

Measuring effective access will contribute to better targeted strategies toward universal health coverage. The analysis presented here highlights a need to improve quality, availability, and opportuneness (location and time) of health services provision in Mexico.

